# Mitochondrial DNA and Epigenetics: Investigating Interactions with the One-Carbon Metabolism in Obesity

**DOI:** 10.1155/2022/9171684

**Published:** 2022-01-29

**Authors:** Laura Bordoni, Irene Petracci, Monika Mlodzik-Czyzewska, Anna M. Malinowska, Artur Szwengiel, Marcin Sadowski, Rosita Gabbianelli, Agata Chmurzynska

**Affiliations:** ^1^Unit of Molecular Biology and Nutrigenomics, University of Camerino, Camerino (MC), Italy; ^2^School of Advanced Studies, University of Camerino, Camerino (MC), Italy; ^3^Department of Human Nutrition and Dietetics, Poznań University of Life Sciences, Poland; ^4^Department of Food Technology of Plant Origin, Poznań University of Life Sciences, Poland

## Abstract

Mitochondrial DNA copy number (mtDNAcn) has been proposed for use as a surrogate biomarker of mitochondrial health, and evidence suggests that mtDNA might be methylated. Intermediates of the one-carbon cycle (1CC), which is duplicated in the cytoplasm and mitochondria, have a major role in modulating the impact of diet on the epigenome. Moreover, epigenetic pathways and the redox system are linked by the metabolism of glutathione (GSH). In a cohort of 101 normal-weight and 97 overweight/obese subjects, we evaluated mtDNAcn and methylation levels in both mitochondrial and nuclear areas to test the association of these marks with body weight, metabolic profile, and availability of 1CC intermediates associated with diet. Body composition was associated with 1CC intermediate availability. Reduced levels of GSH were measured in the overweight/obese group (*p* = 1.3∗10^−5^). A high BMI was associated with lower LINE-1 (*p* = 0.004) and nominally lower methylenetetrahydrofolate reductase (*MTHFR*) gene methylation (*p* = 0.047). mtDNAcn was lower in overweight/obese subjects (*p* = 0.004) and independently correlated with *MTHFR* methylation levels (*p* = 0.005) but not to LINE-1 methylation levels (*p* = 0.086). DNA methylation has been detected in the light strand but not in the heavy strand of the mtDNA. Although mtDNA methylation in the light strand did not differ between overweight/obese and normal-weight subjects, it was nominally correlated with homocysteine levels (*p* = 0.035) and *MTHFR* methylation (*p* = 0.033). This evidence suggests that increased body weight might perturb mitochondrial-nuclear homeostasis affecting the availability of nutrients acting as intermediates of the one-carbon cycle.

## 1. Introduction

Obesity is a complex disease characterized by a low-grade inflammation status determined by an excessive accumulation of dysfunctional adipose tissue [1]. It represents a global health problem since the prevalence of obesity worldwide has reached epidemic proportions [2]. In this pathological condition, nutrient excess or disequilibrium also lead to mitochondrial dysfunctions [3], which may contribute to the increased risk of obesity-driven pathologies [4, 5].

Mitochondria are essential organelles for several aspects of cellular homeostasis, including such things as cellular energy production, regulation of apoptosis, and maintenance of calcium homeostasis [6]. Mitochondria are the only mammalian cellular organelle containing an independent genome, the circular double-stranded mitochondrial DNA (mtDNA) [7], which encompasses only 37 genes, 13 of which encode respiratory chain subunits and 24 of which are tRNA and rRNA components. mtDNA can be found in a different copy number depending on the cell and tissue type, as well as on the cellular health status [8]. Despite mtDNA being essential for mitochondrial functions, many mitochondrial proteins are also encoded by nuclear genes. This keeps mitochondria (and mtDNA) in strict crosstalk with the nuclear DNA [9].

Mitochondrial dynamics are regulated by both intrinsic and extrinsic factors, including nutrition [10]. Indeed, food provides substrates and cofactors for mitochondrial enzymes that are essential in boosting mitochondrial functions [11]. Moreover, nutritional status and the overall body composition contribute to the regulation of epigenetic homeostasis [12], which also affects mitonuclear communication [7, 10, 13, 14]. The crosstalk between mitochondria and the nuclear epigenome is bidirectional: mitochondria mediate epigenetic processes and, conversely, change in the epigenome control mitochondrial functions, thus affecting stress responses and longevity [9, 10, 14]. Mitochondria are crucial to providing intermediate metabolites, which are required for epigenetic modifications in the nucleus, which in turn control the expression of mitochondrial proteins. In particular, the one-carbon cycle (1CC) has a central role in linking metabolic and epigenetic regulations. Folate, vitamin B12, betaine, and choline all contribute to the regulation of the remethylation of homocysteine into methionine, which is used as a methyl-donor (in the form of S-adenosyl-methionine) for methylation reactions in the cell. This metabolic pathway involves several enzymes, among which the methylenetetrahydrofolate reductase (encoded by the *MTHFR* gene) is one of the most important since it regulates cellular levels of 5-methyl-tetrahydrofolate, which is the bioactive form of folate. Expression of the *MTHFR* gene can be regulated by methylation of its promoter [15], which has been shown to vary in obesity [16], as well as in response to environmental stimuli, like smoking [17]. Interestingly, the 1CC is duplicated in the cell, with the mitochondrial and cytosolic 1CCs sharing the previously mentioned intermediates [18]. In addition, endogenous synthesis of glutathione (GSH), which is a major antioxidant, starts from homocysteine, which is converted into GSH through several steps including the transsulfuration pathway [19]. For these reasons, nutrition, the redox system, and epigenetics are strictly interconnected. Previous studies suggested a potential impact of diet on mtDNAcn [20, 21] and also on mtDNA methylation [22, 23]. Indeed, it has recently been demonstrated that DNA methylation occurs not only in the nDNA but also in the mtDNA (especially in the displacement loop or D-loop) [24]. However, the mechanistic effects that it exerts on mtDNA replication and transcription are still discussed [25, 26]. Alterations of mtDNA methylation have been associated with multifactorial diseases such as cardiovascular diseases (CVDs) [27, 28] and obesity [29], as well as with environmental exposures including pollutants [30, 31] and nutrition [22]. For this reason, mtDNA methylation appears as a promising mediator between environmental exposure (i.e., diet) or metabolic conditions (i.e., obesity) and mitochondrial health. In addition, the blood level of mtDNA copy number (mtDNAcn) has emerged as a promising biomarker, especially in obesity [32, 33], metabolic diseases (e.g., type 2 diabetes) [34–36], and CVD [37, 38], and clinical interventions have been shown to modulate this parameter [39]. Despite the link between mtDNA, environmental exposures and complex diseases, and mtDNA's potential role as a biomarker, no previous research projects investigated mtDNAcn in people with increased body weight correlating it with the nutritional status, the abundance of 1CC intermediates, and with mtDNA methylation.

To disentangle the complex picture between nutrition, epigenetics, and mitochondria in overweight and obesity, we studied a group of 198 subjects (101 controls and 97 overweight or obese persons), with several parameters associated with the 1CC and epigenetic homeostasis having been measured, including the intake of nutrients with an epigenetic impact (i.e., folate, betaine, and choline), blood levels of 1CC intermediates (i.e., folate, betaine, choline, vitamin B12, glutathione (GSH), and homocysteine), the methylation levels of nuclear (i.e., *MTHFR* and LINE-1 repeated elements) and mitochondrial (D-loop) DNA, and the mitochondrial DNA copy number. This study is primarily aimed at evaluating mtDNAcn and D-loop methylation levels (in both heavy and light strands) in a group of subjects with a different body weight status, to estimate the association of these marks with body weight, metabolic profile, and availability of 1CC intermediates associated with diet.

## 2. Methods

### 2.1. Study Population

For this study, we used a subset of 198 randomly chosen participants from a group of 421 subjects enrolled in 2016-2018 in Poland, as previously described [40]. The subjects were male and female adults between 20 and 40 years of age (100 with BMI < 25 kg/m^2^ and 100 subjects with BMI ≥ 25 kg/m^2^). The study was implemented in accordance with the Declaration of Helsinki and the Guidelines for Good Clinical Practice and was approved by the Local Ethics Committee at Poznań University of Medical Sciences (No. 966/15). All study participants gave their informed consent.

Recruitment was conducted using online advertisements circulated through social media and a snowball sampling technique. The exclusion criteria included chronic diseases (such as diabetes, metabolic syndrome, cancer, and hypothyroidism); recent dieting or being on a calorie-restriction diet; use of medication known to affect the taste, body weight, lipid profile, or appetite; moderate or heavy smoking (more than one pack per week); shift work; and being pregnant or lactating.

All the procedures were conducted at the Department of Human Nutrition and Dietetics at Poznań University of Life Sciences.

### 2.2. Sample Collection and Body Composition Measurement

Peripheral blood was collected into tubes containing EDTA. Samples for DNA analysis were stored at -20°C for further analysis, whereas samples for blood biomarkers measurement were centrifuged, with plasma stored at -80°C for analysis. Height was measured to the nearest 0.5 cm using a stadiometer WPT 100/200 OW (RadWag, Poznan, Poland). A Bod Pod (Cosmed, Italy) was used to determine body composition in line with the manufacturer's recommended procedure. Body weight was measured to the nearest 0.1 kg, using the calibrated scale included in the Bod Pod. Waist circumference was measured at the midpoint between the lowest rib and the top of the iliac crest using nonelastic tape. Waist-to-hip ratio (WHR) was calculated as waist circumference divided by hip circumference and considered as an index of metabolic risk as previously demonstrated [41].

### 2.3. Nutritional Assessment

Food intake was assessed using three-day food records. Participants were asked to report all food, beverages, and supplements consumed over 3 days. All participants received a printed food diary and attended educational sessions led by a qualified dietician, where they were instructed in food quantity estimation methods. The energy content and nutritional value of the daily food rations were calculated based on food composition tables using the Diet 6.0 computer software (Food and Nutrition Institute, Warsaw, Poland). The intake of choline and betaine were calculated using the USDA Database for the Choline Content of Common Foods [42, 43].

### 2.4. Blood Biomarker Measurement

Plasma concentrations of choline and betaine were determined using ultrahigh-performance liquid chromatography-electrospray ionization mass spectrometry (RP-UHPLC-ESI-MS), as described earlier [44]. Reversed-phase (column with pentafluorophenyl (PFP) stationary phase) ultrahigh-performance liquid chromatography-electrospray ionization mass spectrometry (RP-UHPLC-ESI-MS) analysis was performed using a Dionex UltiMate 3000 UHPLC (Thermo Fisher Scientific, Sunnyvale, CA, USA) coupled to a Bruker maXis impact ultrahigh-resolution orthogonal quadrupole-time-of-flight accelerator (qTOF), equipped with an ESI source, and operated in the positive-ion mode (Bruker Daltonik, Bremen, Germany). The total homocysteine and glutathione (GSH) levels in plasma were measured using high-performance liquid chromatography [45].

Serum folate and vitamin B12 concentrations were estimated using an enzyme-linked immunosorbent assay method (Folic Acid/Vitamin B_9_ ELISA Kit, Elabscience and VB12 (Vitamin B12) ELISA Kit, and Elabscience, respectively), following the manufacturer's directions. Total Hcy concentrations were measured in plasma samples after derivatization using high-performance liquid chromatography (HPLC) with UV detection.

Serum concentrations of total cholesterol, low-density lipoprotein cholesterol (LDL-C), high-density lipoprotein cholesterol (HDL-C), and triglycerides (TGs) were determined using commercial kits (Thermo Fisher Scientific, Waltham, MA, USA) and standard enzymatic methods with a fully automated Konelab 20i analyzer (Thermo Electron Corporation, Vantaa, Finland). The TG/HDL ratio was calculated and used as an index of cardiovascular and metabolic risk as previously described [46, 47].

### 2.5. DNA Extraction and Mitochondrial DNA Copy Number Assessment

Genomic DNA was isolated from frozen blood using a NucleoSpin Blood kit (Macherey–Nagel, Germany). Quantity and purity were determined by Nanodrop 2.0 (Thermo Fisher, USA). All samples were processed under the same conditions and using the same DNA extraction method because variations in the DNA extraction method might affect the evaluation of mtDNAcn. As previously published, relative mtDNAcn quantification was performed by real-time PCR (Biorad CFX96), with nDNA considered as a normalizer [29, 48]. The mitochondrial primers have been previously validated for their specificity (unique amplification of mtDNA) and the absence of coamplified nuclear insertions of mitochondrial origin (NUMTs) [48]. An interrun calibrator sample was used to adjust the results obtained from different amplification plates.

### 2.6. Bisulphite Pyrosequencing for Nuclear and Mitochondrial DNA Methylation Analysis

DNA methylation was assessed by bisulphite pyrosequencing for both nuclear and mitochondrial sequences. Since the circular structure of mtDNA might affect the bisulphite conversion, 600 ng of DNA was digested with BamHI according to the manufacturer instruction. BamHI cut mtDNA in one point, thus linearising it and reducing the risk of false-positive methylation results. Then, DNA was converted with bisulphite using the EZ-96 DNA Methylation-Gold kit (Zymo Research, Orange, USA) according to the manufacturer's instructions. Since it has been reported that DNA methylation in mtDNA might differ between the two strands, mitochondrial DNA methylation was assessed in two areas of the D-loop, one located on the heavy, the other on the light strand, as previously suggested by Vos et al. [49]. The D-loop region is highly relevant to mitochondrial gene transcription and is one of the few regions on mtDNA that is not included in the nuclear DNA as NUMTs [50]. For this reason, analysis of methylation in this area does not require previous isolation of mtDNA from nuclear DNA.

In terms of *MTHFR* methylation, we studied a CpG island in the 5′-untranslated (UTR) region of the *MTHFR* gene spanning from +30 to +184 from the transcription start site, whose methylation levels were found to be inversely correlated with the *MTHFR* gene expression levels [15, 16, 51].

Primers used to amplify the areas of interest (both nuclear and mitochondrial) are shown in table 1 of supplementary materials. PCR amplification was performed using PyroMark PCR kits (Qiagen Inc., Venlo, the Netherlands) in a thermal cycling device (2720 Thermal cycler, Applied Biosystem, Waltham, USA). Amplicons were checked by gel electrophoresis and then pyrosequenced using the PyroMark Q24 device (Qiagen Inc., Venlo, the Netherlands). The efficiency of the bisulphite conversion was assessed using non-CpG cytosine residues within the analyzed sequence. The degree of methylation was defined as the percentage of methylated cytosines over the sum of methylated and unmethylated cytosines.

### 2.7. Statistical Analysis

Statistical analysis was performed by using SPSS (IBM SPSS Statistics for Windows, Version 24.0, USA) and R version 3.5.3 (R Core Team, Vienna, Austria). Data are reported as the mean ± standard deviation unless specified otherwise. The Kolmogorov-Smirnov test was used for the analysis of the normality of data distribution. The *T*-test and Mann–Whitney statistics were used to compare the mean differences between controls and obese subjects for parametric and nonparametric analyses, respectively. Pearson's correlation (*r*) or Spearman's Rho coefficients were measured to test parametric and nonparametric correlations between continuous variables. Multiple linear regression was applied to test associations between continuous variables adjusting the model for covariates. Age and sex were always considered as confounding variables. If other parameters were added as confounding variables to check for independent associations, this is explicitly indicated in the text. Parameters with skewed distributions were appropriately log-transformed before parametric analyses. The varimax-rotated principal component analysis (PCA) was used as a technique for dimension reduction in multivariate analysis. A *p* value < 0.05 was considered significant throughout the study. The Bonferroni correction was applied to correct *p* values in multiple comparisons. Due to the exploratory character of the work, nominal *p* values were also displayed. Technical replicates were not considered for the inference statistics (no inflation of units of analysis was performed).

## 3. Results

### 3.1. Study Population Characteristics

Data of 198 subjects were analyzed; this included 49.5% males and 50.5% females, equally distributed in the normal-weight (*N* = 101) and the overweight/obese groups (*N* = 97). The mean age in these groups was 27 ± 5 and 28 ± 5 years, respectively. Despite statistical significance (*p* = 0.038), this difference is not biologically meaningful, and the two groups can be considered highly comparable for age. Significant differences in body composition and lipid profile were measured between the groups, and detailed descriptive statistics are provided in table 2 of supplementary materials.

### 3.2. Intake and Circulating Levels of 1CC Intermediates in All Subjects

In terms of dietary intake, a comparison of energy, folate, choline, and betaine intake in the two groups is provided in table 3 of supplementary materials. No significant differences were measured between the groups, except for a nominally different lower folate intake in the obese group (*p* = 0.036) (supplementary table 3). Correlations between dietary intakes and body composition are shown in supplementary table 4. A correlation matrix for dietary intake and circulating levels of these nutrients is shown in table 5 of supplementary materials.

No significant differences were measured between cases and controls, either in the circulating folate, betaine, and choline levels or homocysteine levels (Table 1). Vitamin B12 circulating levels were nominally lower in overweight/obese than in the control group (*p* = 0.045). Significantly lower circulating levels of GSH were detected in the overweight/obese group compared to controls (controls: 11.5 ± 3.9 and overweight/obese: 8.9 ± 3.7; *p* = 1.3∗10^−5^) (Table 1).

GSH was inversely correlated with BMI (Spearman′s Rho = −0.321; *p* = 4.3∗10^−6^), WHR (Spearman′s Rho = −0.273; *p* = 1.1∗10^−4^), and FM% (Spearman′s Rho = −0.242; *p* = 0.001). A logistic regression model adjusted for age and sex confirmed that overweight/obese subjects have significantly lower levels of GSH than normal-weight people (*β* = −0.178; *p* = 2.4∗10^−5^). Interestingly, GHS levels were associated with vitamin B12 levels (*β* = 0.160; *p* = 0.029)—even when adjusting the analysis for sex, age, and FM%—suggesting an independent association between these parameters.

**Figure 1:**
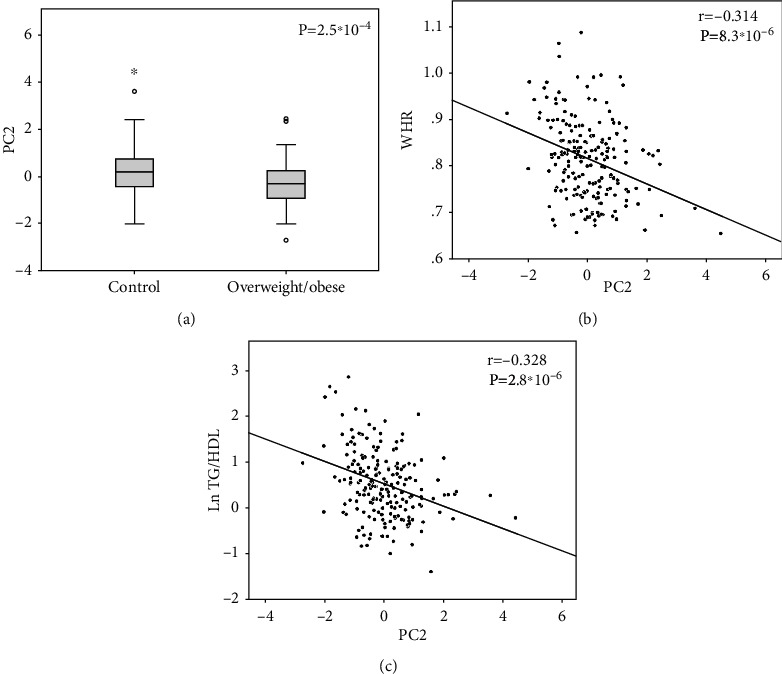
The *T*-test showed different PC2 distribution (a) between the control and the overweight/obese groups. Pearson's correlation showed that PC2 was negatively correlated with (b) WHR and (c) LnTG/HDL in the whole sample. Details on PCs are shown in supplementary table 6. Ln: logarithmic function.

Given the presence of multicollinearity between the analyzed variables, in order to test the associations between epinutrients and body composition, we performed an unsupervised principal component analysis (PCA) of such elements as folate, homocysteine, GSH, betaine, choline, and vitamin B12 (details in supplementary table 6). The *T*-test showed that PC2 (which is mainly determined by high levels of folate, GSH, and vitamin B12 and low levels of homocysteine) was lower in the obese than in the control group (*p* = 2.5∗10^−4^) (Figure 1(a)). Indeed, it was inversely correlated with BMI (*r* = −0.288; *p* = 4.7∗10^−5^), WHR (*r* = −0.314; *p* = 8.3∗10^−6^) (Figure 1(b)), and with FM% (*r* = −0.197; *p* = 0.006). Despite none of the 1CC-associated factors were being correlated with TG/HDL ratio if analyzed singularly, PC2 was highly associated with TG/HDL ratio (*r* = −0.328; *p* = 2.8∗10^−6^) (Figure 1(c)), even when adjusting the analysis for the FM% (*β* = −0.181; *p* = 0.005), suggesting an independent impact of 1CC intermediate availability on this parameter.

### 3.3. *MTHFR* Methylation, Body Composition, Lipid Profile, and 1CC Nutrients

The selected area of *MTHFR* included 2 CpGs, with a mean % level of 32.9 ± 5.3 in the whole group. The mean % methylation of *MTHFR* decreased with age (Spearman′s Rho = −0.170; *p* = 0.018). No significant differences were measured between sex groups (*p* > 0.05). Methylation of *MTHFR* was lower in the overweight/obese individuals than in the normal-weight group considering the first CpG (*p* = 0.045) but not the second (*p* > 0.05). Raw correlations between the mean *MTHFR* methylation, body composition, and circulating 1CC intermediates are shown in supplementary table 7. Upon adjusting the analysis for sex and age, the mean *MTHFR* methylation was nominally associated with BMI (*β* = −0.147; *p* = 0.047) and WHR (*β* = −0.117; *p* = 0.036), but not with FM% (*p* = 0.221).

PC2 was associated with *MTHFR* methylation (adjusting for age and sex; *β* = 0.156; *p* = 0.032). Testing the 1CC intermediates singularly, there were no direct correlations between *MTHFR* methylation and circulating betaine (*p* > 0.05), choline (*p* > 0.05), or vitamin B12 (*p* > 0.05) (supplementary table 7). However, *MTHFR* methylation was nominally correlated with circulating levels of folate (Spearman′s Rho = 0.154; *p* = 0.033) (Figure 1(b)), even after adjusting the analysis for sex and age (*β* = 0.161; *p* = 0.027), suggesting that folate is the major driver of the association between *MTHFR* and PC2.

**Figure 2:**
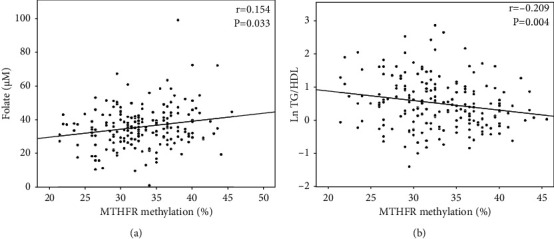
*MTHFR* methylation was positively correlated with (a) folate circulating levels while it was negatively correlated with the (b) TG/HDL ratio. Spearman's Rho and *p* values are shown. Ln: logarithmic function.

MTHFR methylation was negatively correlated with the TG/HDL ratio (Spearman′s Rho = −0.209; *p* = 0.004) (Figure 2(b)). This association was significant even upon adjusting the analysis for sex and age (*β* = −0.196; *p* = 0.005) and FM% (*β* = −0.216; *p* = 0.010).

These findings suggest that high methylation levels are associated with a better metabolic profile in our sample.

### 3.4. mtDNAcn, Body Composition, and 1CC Intermediates

**Figure 3:**
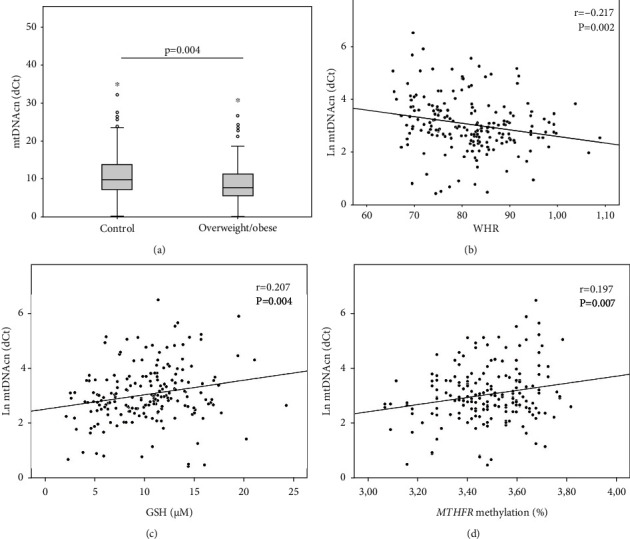
mtDNAcn and body composition. mtDNAcn is lower in (a) overweight/obese than in controls, and it is inversely correlated with (b) WHR. mtDNAcn positively correlates with (c) GSH levels and (d) *MTHFR* methylation. Pearson's correlation and *p* values are shown as well. Ln: logarithmic function.

mtDNAcn was inversely correlated with age (Spearman′s Rho = −0.155; *p* = 0.030). No significant differences were found between males and females (*p* > 0.05). mtDNAcn was lower in the overweight/obese group than in the normal-weight subjects (11.5 ± 7 vs. 9.2 ± 6.6; *p* = 0.004) (Figure 3(a)). mtDNAcn was correlated with WHR (*r* = −0.217; *p* = 0.002) (Figure 3(b)), but not with BMI (*r* = −0.118; *p* > 0.05) or FM% (*r* = −0.053; *p* > 0.05). After adjusting the analysis for age and sex, the correlation between mtDNAcn and WHR was confirmed (*β* = −0.220; *p* = 0.021). No significant correlations were detected between mtDNAcn and any of the blood lipid parameters (supplementary table 8), despite a nominal correlation with TG/HDL being detected (Spearman′s Rho = −0.145; *p* = 0.044).

Concerning circulating levels of 1CC metabolites, PC2, but not PC1, was correlated with mtDNcn (*r* = 0.169; *p* = 0.019). Testing each variable singularly, mtDNAcn was correlated with GSH levels (*r* = 0.207; *p* = 0.004) (Figure 3(c)) but not with vitamin B12 (*p* = 0.075), folate (*p* > 0.05), betaine (*p* > 0.05), or choline (*p* > 0.05). The correlation between mtDNAcn and WHR remained significant even after the adjustment for GSH (*β* = −0.166; *p* = 0.021), suggesting an independent association between mtDNAcn and body composition.

Remarkably, mtDNAcn was also associated with *MTHFR* methylation levels (*r* = 0.197; *p* = 0.007) (Figure 3(d)). A linear regression model, adjusted for GSH, age, sex, and FM%, confirmed an interesting independent association between mtDNAcn and *MTHFR* methylation levels (*β* = 0.166; *p* = 0.022). GSH was confirmed as a confounder of this association (*β* = 0.181; *p* = 0.018) but FM% was not (*p* > 0.05). Overall, this evidence corroborates the hypothesis of a link existing between mitochondrial dynamics and epigenetic homeostasis, independently of the body composition.

### 3.5. mtDNA Methylation

Barely detectable methylation levels were measured in the D-loop area when analyzing the heavy chain in our study group (1.25 ± 0.67%). On the other hand, higher methylation levels were detected in the light strand of the D-loop (LDLR2) area (4.66 ± 1.21%). Thus, we considered only LDLR2 methylation for further analyses. Among the 3 CpGs analyzed in this area, the first one showed higher net % values (7.6 ± 1.8) than the second (3.0 ± 1.1) and the third CpG (3.0 ± 1.2), thus providing the major contribution to the mean methylation levels. Adjusting the analysis for age and sex, LDLR2 methylation did not show a significant correlation with mtDNAcn (*β* = 0.122; *p* > 0.05). LDLR2 methylation levels did not differ per se between the normal-weight (4.7 ± 1.1%) and the overweight/obese (4.6 ± 1.3%) subjects (*p* > 0.05). LDLR2 was not significantly correlated with the BMI (*p* > 0.05), WHR (*p* > 0.05), FM% (*p* > 0.05), or lipid profile (supplementary table 9) in the whole group.

**Figure 4:**
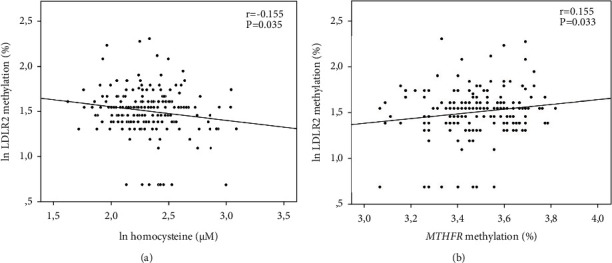
*LDLR2* methylation correlations with (a) homocysteine levels and (b) *MTHFR* methylation levels. Pearson's correlations and *p* values are shown. Ln: logarithmic function.

No significant associations between LDLR2 methylation and PC1 (*p* > 0.05) or PC2 (*p* > 0.05) were detected. Testing the variables singularly (supplementary table 9), LDLR2 was nominally correlated only with blood homocysteine concentrations (*r* = −0.155; *p* = 0.035) (Figure 4(a)).

Surprisingly, LDLR2 methylation levels were linearly correlated with *MTHFR* methylation (*r* = 0.155; *p* = 0.033) (Figure 4(b)). To check if the association between methylation in the LDLR2 and *MTHFR* was mediated by the 1CC, we tested a linear regression model adjusted for age, sex, folate, homocysteine, vitamin B12, and GSH levels. The association between LDLR2 and *MTHFR* methylation was confirmed as independent (*β* = 0.181; *p* = 0.019). Only homocysteine appeared, as expected, as a confounder of this association and was inversely correlated with LDLR2 methylation (*β* = −0.170; *p* = 0.028).

### 3.6. LINE-1 Methylation

**Figure 5:**
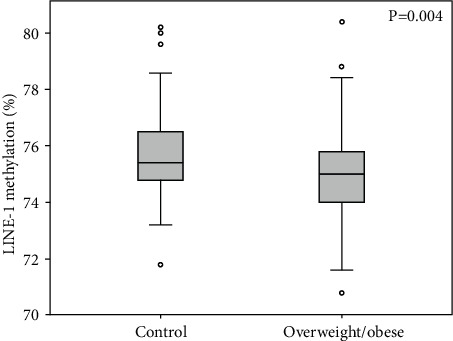
LINE-1 methylation levels are significantly lower in the overweight/obese than in the control group. The *p* value from the *T*-test is also indicated.

The LINE-1 methylation level was 75.49 ± 1.7% in the whole group. LINE-1 was nominally correlated with age in the healthy (Spearman′s Rho = −0.226; *p* = 0.026) but not in the overweight/obese group (Spearman′s Rho = 0.089; *p* > 0.05). The mean LINE-1 methylation levels were significantly lower in the overweight/obese (75.04 ± 1.67%) than in the controls (75.74 ± 1.65%) (*p* = 0.004) (Figure 5). Also, a logistic regression model adjusted for sex and age confirmed that subjects from the overweight/obese group had a higher probability to have lower LINE-1 methylation levels with respect to the normal-weight subjects (OR = 0.775; *p* = 0.008). Indeed, LINE-1 methylation levels were negatively correlated with BMI (*r* = −0.209; *p* = 0.004) and FM% (*r* = −0.160; *p* = 0.028). No significant correlation with WHR (*r* = −0.092; *p* = 0.209) was measured. Upon adjusting the analysis for age and sex, the results confirmed the association of LINE-1 methylation levels with BMI (*β* = −0.198; *p* = 0.007) and FM% (*β* = −0.230; *p* = 0.008), but not with WHR (*β* = −0.091; *p* > 0.05). The associations with BMI (*β* = −0.180; *p* = 0.021) and FM% (*β* = −0.215; *p* = 0.022) were confirmed also after adjusting the analysis for plasma GSH concentration, which was not a confounder in these associations.

LINE-1 methylation was associated with PC2 (*β* = 0.187; *p* = 0.012), but this association was no more significant when adjusting the analysis for FM% (*β* = 0.132; *p* > 0.05). Testing the 1CC intermediates singularly, only vitamin B12 circulating levels are nominally associated with this parameter (*β* = 0.182; *p* = 0.019), even adjusting the analysis for the FM% (*β* = 0.156; *p* = 0.044), suggesting an independent contribution of this parameter on LINE-1 methylation. LINE-1 methylation was nominally associated with mtDNAcn (*β* = 0.148; *p* = 0.046); however, this was no longer significant after adjusting the analysis for the FM% (*β* = 0.126; *p* > 0.05) which is a significant confounder in this association (*β* = −0.207; *p* = 0.018).

No significant correlations were detected between LINE-1 and *MTHFR* upon either adjusting for age and sex (*β* = 0.044; *p* > 0.05) or adding FM% to the model (*β* = 0.025; *p* > 0.05). Similarly, LDLR2 methylation was not associated with LINE-1 when adjusting for age and sex (*β* = 0.096; *p* > 0.05) and FM% (*β* = 0.108; *p* > 0.05).

## 4. Discussion

In our study, low levels of 1CC's intermediates (GSH, vitamin B12, and folate) have been associated with worse body composition, lipid profile, and changes in DNA methylation. The association between 1CC markers and lipid metabolism has been discussed in previous papers [44, 45]. In terms of GSH, there is evidence suggesting that oxidative stress—particularly the reactive oxygen species (ROS) imbalance in adipose tissue and consequential adipokines' release—may be the mechanistic link between obesity and its associated metabolic complications [52]. Since GSH is a major antioxidant, it has been suggested that it plays a role in preventing the onset of obesity and its comorbidities (e.g., type II diabetes and CVD). However, several findings have shown dual and seemingly contradictory roles of GSH in obesity [53]. Alterations of GSH and 1CC intermediates have been previously shown, even though the direction of the association is yet to be determined. For example, in an animal model, methionine restriction reduced GSH levels in the liver (50%) and kidney (40%) but nearly doubled levels of GSH in whole blood, suggesting elevated secretion of GSH as a source of cysteine [54–56]. On the other hand, folic acid supplementation in obese individuals has been demonstrated to increase GSH levels, which is indicative of a reduction in oxidative stress [57].

In our study, GSH was associated not only with better body composition but was also directly correlated with mtDNAcn, especially if associated with impairments of other 1CC intermediates (PC2). This is consistent with previous findings showing that depletion of mtDNAcn decreased GSH in myoblasts, suggesting that ROS homeostasis and antioxidant enzymes are modulated by the cellular mtDNA content [58]. Consistently, a reduction of mtDNAcn in leukocytes has been previously associated with the oxidative stress in blood circulation elicited by the alteration of plasma antioxidants/prooxidants [59]. These findings corroborate the hypothesis that the deregulation of the oxidative system induced by obesity might also affect mtDNAcn. Indeed, the mtDNA content can be adjusted according to metabolic needs, and mtDNA expression is a prerequisite for the biogenesis of the OXPHOS system.

Interestingly, mtDNAcn was lower in the overweight/obese group than in the controls in our study. A reduction of mtDNAcn is consistent with the reduced metabolic flexibility reported in obesity [60, 61] and might contribute to explaining why the shift to *β*-oxidative metabolism is impaired in these subjects. It has been shown that obesity-induced low-grade inflammation reduces energy generation and expenditure in adipocyte mitochondria, further aggravating obesity, establishing a positive-feedback loop, and increasing fat accumulation and weight gain [62]. Indeed, lower baseline mtDNAcn has been prospectively associated with a higher risk of type 2 diabetes [63]. Moreover, the reduction of mtDNAcn (both measured in leucocytes [37, 38] or whole blood [64]) has been recently shown to associate with CVD, thus representing a deregulated pathway that might contribute to explaining the comorbidity of these two complex pathologies.

Given the relevance of mtDNAcn and its plastic regulation, we also tested if circulating levels of other 1CC intermediates might be associated with a different mtDNAcn in our population. However, no other significant associations (in addition to GSH) were detected. In particular, no associations were detected between mtDNAcn and folate levels, despite the role of mitochondrial folate in supporting mtDNA synthesis [13] and despite its deficiency having been previously correlated with the accumulation of mtDNA deletions and reduced mtDNA content in animal models [65].

On the other hand, mtDNAcn was associated with certain important methylation marks. In particular, it linearly correlated with methylation levels in the *MTHFR* promoter. Methylation in this area was, per se, associated with circulating folate levels and negatively correlated with the WHR and the TG/HDL ratio. Interestingly, the association between mtDNAcn and *MTHFR* methylation was confirmed even after adjusting the analysis for confounders such as folate levels, GSH, or body weight status. It is not the first time that mtDNAcn is associated with different methylation levels in the nuclear DNA [66–69]. Previous findings demonstrated that mtDNAcn affects nuclear DNA methylation at specific loci, resulting in gene expression changes that may impact human health and disease via altered cell signaling [69]. Thus, an interesting focus on mtDNAcn, methyl-metabolism, and DNA methylation emerged from this study. Nevertheless, the mechanistic effect of DNA methylation in this area of the *MTHFR* gene expression is not completely clear. Previous studies showed that increased methylation was associated with suppression of gene expression [15] and disease conditions [15, 70, 71]. On the other hand, recent findings associated a reduction of *MTHFR* methylation with unfavorable conditions or diseases. For example, hypermethylation of the *MTHFR* promoter has been described as a protective factor against ischemic stroke [72]. Moreover, it has been demonstrated that *MTHFR* demethylation is more frequent in subjects that are physically inactive [73], and that homocysteine can induce demethylation in the promoter region of the *MTHFR* gene in human vascular smooth muscle cells [74]. Recent studies have shown that *MTHFR* promoter methylation is also linked to circulating folate, vitamin B12, and homocysteine levels in individuals affected by Alzheimer's [75] and cardiovascular diseases [76], suggesting that the *MTHFR* methylation status could be a mediator of impairments of the folate metabolic pathway. Our results support the hypothesis that higher methylation levels in this area are not necessarily detrimental, since they are associated with better body composition and a reduced TG/HDL ratio, which is a well-known risk factor for cardiovascular diseases [46, 47]. Moreover, we detected a positive correlation between folate levels and *MTHFR* methylation, similarly to what has been previously reported [75, 76], suggesting that lower *MTHFR* promoter methylation might reflect the need of promoting its transcription in case of low folate availability (which might occur in overweight/obese individuals [45]). However, further mechanistic studies on the effect of DNA methylation in this area on *MTHFR* expression and its impact on health are necessary to draw a final conclusion.

Interestingly, LINE-1 methylation was lower in overweight/obese subjects in our study, showing a strong correlation with FM%. Independently of age, we can confirm that increased body weight is associated with demethylation of LINE-1 even in young adults (aged 20-40). This is consistent with previous findings showing a reduction of this mark in the adipose tissue of obese subjects [77], despite contrasting evidence from measures of the same parameter in blood [78]. LINE-1 methylation levels have previously been associated with genome instability and ageing [79]. Remarkably, LINE-1 methylation levels have been proposed as a biomarker of weight loss in obese subjects, influenced by dietary antioxidant capacity [80]. In our study, LINE-1 methylation was nominally associated with the availability of circulating B12, demonstrating the relevance of this vitamin for epigenetic homeostasis and genome stability, as previously hypothesized [81]. This methylation mark was not directly associated with methylation in the *MTFHR* gene, despite both being lower in the overweight/obese group than in the control group. A mild correlation of this parameter with mtDNAcn was also detected, but it seems to be mediated by confounders like overweight (which is independently associated with mtDNAcn).

In terms of methylation in the mtDNA, we detected very low levels in the heavy strand, where methylation was barely detectable. Higher levels were measured in the light strand. This is consistent with previous findings showing higher methylation in the light than in the heavy strand [49, 82]. No significant associations of this epigenetic mark with body composition were detected. Despite LDLR2 methylation not being associated with LINE-1 methylation, it was independently correlated with *MTHFR* methylation. This was also confirmed after adjusting the model for the circulating levels of 1CC intermediates, with homocysteine being an independent confounder in this association. This evidence suggests a functional role of mtDNA methylation, even though a mechanistic explanation has not been described yet. Despite some interesting findings that have emerged, the topic of DNA methylation in mtDNA continues to be tricky and its biological role remains to be elucidated.

A limitation of this study is that molecular marks (especially epigenetic alterations) have been measured in DNA extracted from whole blood; however, they can also be cell-specific. This might explain why the strength of several associations is not high. However, this represents the first attempt to investigate mtDNA, epigenetics marks, and intermediates at a systemic level. Further studies are necessary to define causal and mechanistic aspects of the measured associations.

## Figures and Tables

**Table 1:** Blood levels of 1C metabolism intermediates, related to nutrition, in the two groups (control vs. obese). The *T*-test was used to identify significant differences between the groups. Hcy: homocysteine; GSH: glutathione.

	Controls (*N* = 101)				Overweight/obese (*N* = 97)				*p*
	Min	Max	Mean	SD	Min	Max	Mean	SD	
Hcy (*μ*M)	5.58	29.95	10.64	3.55	5.07	21.95	10.71	3.15	>0.05
GSH (*μ*M)	2.60	24.26	11.52	3.94	2.18	17.74	8.97	3.68	1.3∗10^−5^
Folate (ng/mL)	9.72	98.76	37.12	12.99	1.23	77.32	34.85	11.87	>0.05
Betaine (*μ*M)	8.92	110.29	38.09	14.62	7.77	75.93	35.07	12.85	>0.05
Choline (*μ*M)	3.54	10.76	6.10	1.35	3.20	11.01	6.32	1.47	>0.05
Vitamin B12 (*μ*M)	0.32	16.49	2.34	1.96	0.46	13.10	2.08	1.82	**0.045**

## Data Availability

The data used to support the findings of this study are available from the corresponding author upon request.

## Supplementary Materials

Supplementary Materials Supplementary materials contain additional information about primers used for the assays, details about PCA analysis and correlation matrices, and tables that are not fully displayed in the main text.
