# Supplementary material for: Mitochondrial DNA and Epigenetics: Investigating Interactions with the One-Carbon Metabolism in Obesity
Source: Oxid Med Cell Longev. 2022 Jan 29;2022:9171684. doi: 10.1155/2022/9171684 (PMC8817841; doi:10.1155/2022/9171684)
Supplement: Supplementary Materials — contain additional information about primers used for the assays, details about PCA analysis and correlation matrices, and tables that are not fully displayed in the main text. [file 9171684.f1.docx]

**SUPPLEMENTARY MATERIALS**

**Supplementary materials Table 1.** Primer sets used for DNA methylation analysis by pyrosequencing in the different areas of mitochondrial or nuclear DNA.

|  | **Forward (5'-3')** | **BIO-Reverse (5'-3')** | **Sequencing (5'-3')** |
| --- | --- | --- | --- |
| **D-LOOP (heavy strand)** | TGGAAAGTGGTTGTGTAGATATTTAA | CTTTAATTCCTACCTCATCCTATTATTT | AATTAATTAATATATTTTAGTAAG |
| **LDLR2 (light strand)** | TTTTTAGTGTATTGTTTTGAGGAGGTAAGT | CACTCCCATACTACTAATCTCATCA | TTTTTGGGGTTTGGT |
| ***MTHFR*** | TTTTAATTTTTGTTTGGAGGGTAGT | AAAAAAACCACTTATCACCAAATTC | TGAGAGTTTTAAAGATAGTT |
| **LINE-1** | TTTTGAGTTAGGTGTGGGATATA | AAAATCAAAAAATTCCCTTTC | AGTTAGGTGTGGATATAGT |

**Supplementary materials Table 2.** Descriptive statistics of the whole group (N=198) and subgroups divided by body weight status.

|  | All subjects (N=198) | | | | Normal weight (N=101) | | | | Overweight/Obese (N=97) | | | |  |
| --- | --- | --- | --- | --- | --- | --- | --- | --- | --- | --- | --- | --- | --- |
|  | Min | Max | Mean | SD | Min | Max | Mean | SD | Min | Max | Mean | SD | **P** |
| Age (y/o) | 20 | 40 | 28.04 | 5.3 | 21 | 40 | 27.50 | 5.39 | 20 | 40 | 28.65 | 5.17 | **0.038** |
| BMI (kg/m^2^) | 17.1 | 51.11 | 26.02 | 5.2 | 17.1 | 24.98 | 22.14 | 1.87 | 25.07 | 51.11 | 30.05 | 4.43 | **5.5*10^-34^** |
| WHR | 0.66 | 1.09 | 0.82 | 0.1 | 0.66 | 1.09 | 0.78 | 0.07 | 0.69 | 1.06 | 0.85 | 0.08 | **2.5*10^-10^** |
| FM (%) | 7.7 | 60 | 29.47 | 10.8 | 7.7 | 41.8 | 23.13 | 0.76 | 12.5 | 60 | 36.07 | 9.50 | **7.6*10^-18^** |
| Cholesterol (mg/dL) | 63 | 266.9 | 179.92 | 33.2 | 63 | 247.96 | 174.70 | 31.55 | 119.98 | 266.9 | 186.00 | 33.66 | **0.027** |
| HDL-C (mg/dL) | 22.73 | 113.2 | 56.42 | 16.3 | 34.24 | 113.2 | 60.99 | 15.85 | 22.73 | 105.95 | 51.80 | 15.42 | **5.6*10^-5^** |
| LDL-C (mg/dL) | 46.37 | 178.11 | 103.75 | 27.2 | 46.37 | 167.04 | 97.78 | 25.63 | 54.55 | 178.11 | 110.41 | 27.25 | **0.001** |
| TG (mg/dL) | 18.87 | 500.44 | 105.37 | 66.9 | 18.87 | 266.63 | 83.81 | 40.29 | 38.9 | 500.44 | 128.18 | 80.79 | **4.7*10^-6^** |
| TG/HDL | 0.25 | 17.33 | 2.26 | 2.28 | 0.25 | 6.18 | 1.55 | 1.06 | 0.54 | 17.33 | 3.00 | 2.90 | **6.8*10^-8^** |

BMI: body mass index; WHR: waist-to-hip ratio; FM: fat mass; HDL-C: high-density lipoproteins cholesterol; LDL-C: low-density lipoprotein cholesterol; TG: triglycerides.
P Bonf=0.006

**Supplementary materials Table 3.** Comparison of energy, folate, choline and betaine intake in the two groups.

| **Daily intakes** | Controls (N=101) | | | | Obese (N=97) | | | |  |
| --- | --- | --- | --- | --- | --- | --- | --- | --- | --- |
|  | Min | Max | Mean | SD | Min | Max | Mean | SD | P |
| Energy (kcal/d) | 1162.63 | 3933.53 | 2133.31 | 627.04 | 498.32 | 4017.66 | 2070.17 | 690.43 | 0.537 |
| Folate (µg) | 119.01 | 1466.43 | 375.45 | 241.89 | 78.99 | 1807.27 | 315.39 | 210.51 | **0.036** |
| Choline (mg) | 133.6 | 2217.05 | 465.19 | 273.74 | 100.09 | 1579.32 | 439.40 | 232.33 | 0.484 |
| Betaine (mg) | 11.64 | 545.3 | 125.34 | 94.78 | 3 | 516.79 | 122.93 | 105.55 | 0.527 |
| p bonf=0.0125 |  |  |  |  |  |  |  |  |  |

Note: for a more extensive discussion of dietary intakes and body composition the reader can refer to Młodzik-Czyżewska MA et al. (Mlodzik-Czyzewska MA, Malinowska AM, Chmurzynska A. Low folate intake and serum levels are associated with higher body mass index and abdominal fat accumulation: a case control study. Nutr J. 2020 Jun 4;19(1):53. doi: 10.1186/s12937-020-00572-6. PMID: 32498709; PMCID: PMC7273685.)

**Supplementary materials Table 4.** Correlation between dietary intake and body composition.

|  | | Folate intake | Choline intake | Betaine intake | BMI | FM% | WHR |
| --- | --- | --- | --- | --- | --- | --- | --- |
| Folate intake | Spearman's Rho | 1 |  |  |  |  |  |
|  | p | . |  |  |  |  |  |
|  |  |  |  |  |  |  |  |
| Choline intake | Spearman's Rho | 0.521^**^ | 1 |  |  |  |  |
|  | p | **9.5*10^-15^** | . |  |  |  |  |
|  |  |  |  |  |  |  |  |
| Betaine intake | Spearman's Rho | 0.235^**^ | 0.279^**^ | 1 |  |  |  |
|  | p | .001 | **8.4*10^-5^** | . |  |  |  |
|  |  |  |  |  |  |  |  |
| BMI | Spearman's Rho | -0.101 | 0.012 | -0.061 | 1 |  |  |
|  | p | 0.165 | 0.864 | 0.399 | . |  |  |
|  |  |  |  |  |  |  |  |
| FM% | Spearman's Rho | -0.305^**^ | -0.277^**^ | -0.249^**^ | 0.665^**^ | 1 |  |
|  | p | **1.7*10^-5^** | **9.6*10^-5^** | **4.8*10^-4^** | **1.1*10^-26^** | . |  |
|  |  |  |  |  |  |  |  |
| WHR | Spearman's Rho | -0.052 | 0.054 | 0.025 | 0.508^**^ | 0.015 | 1 |
|  | p | 0.479 | 0.458 | 0.732 | **2.5*10^-14^** | 0.830 | . |

P Bonf= 0.002; BMI: body mass index; WHR: waist-to-hip ratio; FM: fat mass.

**Supplementary materials Table 5.** Correlation between circulating levels of nutrients involved in 1CC and 1CC intermediates.

|  |  | Folate intake | Choline intake | Betaine intake | Plasma Hcy | Plasma GSH | Plasma Folate | Plasma Betaine | Plasma Choline | Plasma vitamin B12 |
| --- | --- | --- | --- | --- | --- | --- | --- | --- | --- | --- |
| Folate intake | Spearman's Rho | 1 |  |  |  |  |  |  |  |  |
|  | p | . |  |  |  |  |  |  |  |  |
|  |  |  |  |  |  |  |  |  |  |  |
| Choline intake | Spearman's Rho | 0.521** | 1 |  |  |  |  |  |  |  |
|  | p | **9.4*10^-15^** | . |  |  |  |  |  |  |  |
|  |  |  |  |  |  |  |  |  |  |  |
| Betaine intake | Spearman's Rho | 0.235** | .279** | 1 |  |  |  |  |  |  |
|  | p | **0.001** | **8.4*10^-5^** | . |  |  |  |  |  |  |
|  |  |  |  |  |  |  |  |  |  |  |
| Plasma Hcy | Spearman's Rho | -0.099 | -0.03 | 0.037 | 1 |  |  |  |  |  |
|  | p | 0.175 | 0.679 | 0.615 | . |  |  |  |  |  |
|  |  |  |  |  |  |  |  |  |  |  |
| Plasma GSH | Spearman's Rho | 0.211** | 0.123 | 0.076 | -0.019 | 1 |  |  |  |  |
|  | p | *0.003* | 0.089 | 0.294 | 0.794 | . |  |  |  |  |
|  |  |  |  |  |  |  |  |  |  |  |
| Plasma Folate | Spearman's Rho | 0.108 | 0.03 | 0.108 | -0.099 | 0.096 | 1 |  |  |  |
|  | p | 0.136 | 0.684 | 0.134 | 0.167 | 0.178 | . |  |  |  |
|  |  |  |  |  |  |  |  |  |  |  |
| Plasma Betaine | Spearman's Rho | 0.162* | 0.189** | 0.078 | 0.125 | -0.166* | 0.001 | 1 |  |  |
|  | p | 0.025 | *0.009* | 0.282 | 0.08 | 0.020 | 0.993 | . |  |  |
|  |  |  |  |  |  |  |  |  |  |  |
| Plasma Choline | Spearman's Rho | 0.054 | 0.151* | 0.075 | 0.099 | -0.076 | -0.126 | 0.419** | 1 |  |
|  | p | 0.458 | 0.037 | 0.303 | 0.166 | 0.285 | 0.076 | **7.9*10^-10^** | . |  |
|  |  |  |  |  |  |  |  |  |  |  |
| Plasma vitamin B12 | Spearman's Rho | -0.067 | -0.003 | -0.222** | -0.093 | 0.242** | -0.072 | -0.202** | -0.087 | 1 |
|  | p | 0.36 | 0.967 | *0.002* | 0.195 | **0.001** | 0.317 | *0.005* | 0.228 | . |

Bonf. P=0.001; GSH: glutathione; Hcy: homocysteine.

**Supplementary materials Table 6. Principal component analysis (PCA) identified 2 PC with eigenvalues higher than 1.** A) Description of the individual contribution of the parameters to each PC. Variables showing higher coefficients (either positive or negative) are those that mostly contribute to the PC. B) % Variance and cumulative variance explained by the two PCs.

| **A** | |  | |  |
| --- | --- | --- | --- | --- |
|  | | **PC1** | | **PC2** |
| Plasma Hcy | | 0.353 | | -0.398 |
| Plasma GSH | | -0.041 | | 0.59 |
| Plasma Folate | | 0.115 | | 0.723 |
| Plasma Betaine | | 0.825 | | 0.051 |
| Plasma Choline | | 0.773 | | -0.129 |
| Plasma vitamin B12 | | -0.233 | | 0.386 |
| Varimax rotate matrix, Kaiser normalisation  GSH: glutathione; Hcy: homocysteine; PC: principal component | | | | |
|  |  | |  | |
| **B** |  | |  | |
|  | % variance explained | | % cumulative variance explained | |
| PC1 | 24.528 | | 24.528 | |
| PC2 | 19.943 | | 44.471 | |

The results showed that two principal components (PC), cumulatively explaining 44.5% of the variance, were associated with body composition. In particular, PC1 (to which high levels of choline and betaine mainly contribute) was directly correlated with WHR (Pearson’s correlation=0.296; p=2.7*10-5) but inversely correlated with FM% (Pearson’s correlation=-0.227; p=0.001). However, the association between body composition, betaine (p=0.124) and choline (p=0.793) was not confirmed when testing the associations singularly.

**Supplementary materials Table 7.** Correlations between methylation levels in the analyzed *MTHFR* region and body composition, lipid profile, as well as 1CC intermediate circulating levels.

|  | Spearman's Rho  MTHFR methylation (%) | P value |
| --- | --- | --- |
| BMI | -0.153* | **0.035** |
| Fat mass (%) | -0.085 | 0.245 |
| WHR | -0.11 | 0.129 |
| Total-C | -0.068 | 0.349 |
| HDL-C | 0.184* | **0.011** |
| LDL-C | -0.129 | 0.076 |
| TG | -0.190** | **0.008** |
| TG/HDL | -0.209 | **0.004** |
| Hcy | 0.094 | 0.199 |
| GSH | 0.134 | 0.066 |
| Folate | 0.154* | **0.033** |
| Betaine | 0.069 | 0.347 |
| Choline | -0.044 | 0.548 |
| B12 | -0.004 | 0.955 |

P Bonf=0.004; BMI: body mass index; WHR: waist-to-height ratio;
FM: fat mass; GSH: glutathione; Hcy: homocysteine

**Supplementary materials Table 8. Correlations between mtDNAcn and body composition, lipid profile and 1CC intermediates.**

|  | Spearman's Rho  mtDNAcn (dCt) | P value |
| --- | --- | --- |
| BMI | -0.171^*^ | *0.018* |
| FM% | -0.065 | 0.370 |
| WHR | -0.260^**^ | **2.6*10^-4^** |
| Total-C | 0.038 | 0.604 |
| HDL-C | 0.111 | 0.124 |
| LDL-C | 0.013 | 0.856 |
| TG | -0.147^*^ | *0.041* |
| TG/HDL | -0.145 | *0.044* |
| Hcy | -0.023 | 0.753 |
| GSH | 0.220^**^ | **0.002** |
| Folate | 0.061 | 0.396 |
| Betaine | 0.054 | 0.459 |
| Choline | -0.105 | 0.146 |
| B12 | 0.174^*^ | *0.015* |

P Bonf=0.004; BMI: body mass index; WHR: waist-to-height ratio;
FM: fat mass; GSH: glutathione; Hcy: homocysteine

**Supplementary materials Table 9. Correlations between LDLR2 methylation and body composition, lipid profile and 1CC intermediates.**

|  | | Pearson’s correlation  LDLR2 methylation (%) | P value |
| --- | --- | --- | --- |
| BMI |  | 0.057 | 0.436 |
| FM% |  | 0.087 | 0.237 |
| WHR |  | -0.058 | 0.430 |
| Total-C |  | 0.002 | 0.978 |
| HDL-C |  | 0.117 | 0.110 |
| LDL-C |  | 0.016 | 0.828 |
| TG |  | -0.090 | 0.221 |
| TG/HDL |  | -0.062 | 0.394 |
| Hcy |  | -0.155^*^ | **0**.**035** |
| B12 |  | 0.031 | 0.673 |
| Folate |  | -0.085 | 0.248 |
| Betaine |  | -0.033 | 0.651 |
| Choline |  | 0.002 | 0.978 |
| GSH |  | 0.013 | 0.859 |

BMI: body mass index; WHR: waist-to-height ratio; FM: fat mass;
GSH: glutathione; Hcy: homocysteine; HDL-C: high-density lipoproteins cholesterol;
LDL-C: low density lipoprotein cholesterol; TG: triglycerides; P Bonf=0.004
